# PROSE: Prospective Randomized Trial of the On-X Mechanical Prosthesis and the St Jude Medical Mechanical Prosthesis Evaluation: Part 1(Patient Dynamics): Preoperative demographics and preoperative and operative risk factors

**DOI:** 10.1186/s13019-021-01632-6

**Published:** 2021-11-03

**Authors:** W. R. Eric Jamieson, John L. Ely, Johan Brink, Timothy Pennel, Paul Bannon, Jashvant Patel, Rajiv Kumar Gupta, Prasanna Simha Mohan Rao, Damyanti Agrawal, Lars Wiklund, A. Pieter Kappetein, Rune Haaverstad, Thomas Geisner, Torsten Doenst, Christian Schlensak, Salgunan Nair, Craig Brown, Matthias Siepe, Ralph J. Damiano, Yves Langlois, K. M. Cherian, Hormoz Azar, John C. Chen, Joseph E. Bavaria, Lynn M. Fedoruk, Nabil A. Munfakh, V. Sridhar, Peter M. Scholz, Thomas A. Pfeffer, Jian Ye

**Affiliations:** 1 Vancouver Coastal Health Research Institute, University of British Columbia, Vancouver, Canada; 2 Heart of the Matter CV Consulting, Austin, USA; 3 University of Cape Town, Cape Town, South Africa; 4 University of Sydney, Sydney, Australia; 5 Mehta Mahavir Heart Institute, Surat, India; 6 Dayanand Medical College, Punjab, India; 7 Sri Jayadeva Institute of Cardiovascular Sciences, Bengaluru, India; 8 Banaras Hindu University, Varanasi, India; 9 Sahlgrenska University Hospital, Gothenburg, Sweden; 10 Erasmus University Rotterdam, Rotterdam, Netherlands; 11 Haukeland University Hospital, Bergen, Norway; 12 University Hospital Jena, Jena, Germany; 13 University Hospital Tuebingen, Freiburg, Germany; 14 Apollo Hospital Chennai, Chennai, India; 15 Horizon New Brunswick Heart Center, St. John, Canada; 16 University Heart Center Freiburg, Freiburg, Germany; 17 Washington University School of Medicine, St. Louis, USA; 18 Jewish General Hospital, Montreal, Canada; 19 Frontier Lifeline Hospitals, Chennai, India; 20 Sentara Norfolk General Hospital, Norfolk, UK; 21 Kaiser Permanente Hospital, Honolulu, USA; 22 University of Pennsylvania, Philadelphia, USA; 23 Victoria Heart Institute, Victoria, Canada; 24 Apollo Mulitspecialty Hospitals, Madurai, India; 25 Robert Wood Johnson School of Medicine, New Brunswick, USA; 26 Southern California Permanente Medical Group, Los Angeles, USA

**Keywords:** Mechanical prostheses (demographics and risk factors), Experience Western world and Developing world

## Abstract

**Objectives:**

The PROSE trial purpose is to investigate whether the incidence of thromboembolic—related complications is reduced with a current generation mechanical prosthesis (On-X Life Technologies/CryoLife Inc.—On-X) compared with a previous generation mechanical prosthesis (St Jude Medical—SJM). The primary purpose of the initial report is to document the preoperative demographics, and the preoperative and operative risk factors by individual prosthesis and by Western and Developing populations.

**Methods:**

The PROSE study was conducted in 28 worldwide centres and incorporated 855 subjects randomized between 2003 and 2016. The study enrollment was discontinued on August 31, 2016. The preoperative demographics incorporated age, gender, functional class, etiology, prosthetic degeneration, primary rhythm, primary valve lesion, weight, height, BSA and BMI. The preoperative and operative evaluation incorporated 24 risk factors.

**Results:**

The total patient population (855) incorporated On-X population (462) and the St Jude Medical population (393). There was no significant difference of any of the preoperative demographics between the On-X and SJM groups. The preoperative and operative risk factors evaluation showed there was no significant difference between the On-X and St Jude Medical populations. The preoperative and operative risk factors by valve position (aortic and mitral) also documented no differentiation. The dominant preoperative demographics of the Western world population were older age, male gender, sinus rhythm, aortic stenosis, congenital aortic lesion, and mitral regurgitation. The dominant demographics of the Developing world population were rheumatic etiology, atrial fibrillation, aortic regurgitation, mixed aortic lesions, mitral stenosis and mixed mitral lesions. The Developing world group had only one significant risk factor, congestive heart failure. The majority of the preoperative and operative risk factors were significant in the Western world population.

**Conclusions:**

The preoperative demographics do not differentiate the prostheses but do differentiate the Western and Developing world populations. The preoperative and operative risk factors do not differentiate the prostheses BUT do differentiate the Western and Developing world populations.

**Supplementary Information:**

The online version contains supplementary material available at 10.1186/s13019-021-01632-6.

## Introduction

The purpose of the PROSE (Prospective Randomized Trial of the On-X Mechanical Prosthesis and the St Jude Medical Mechanical Prosthesis Evaluation) study is to investigate whether the incidence of thromboembolic-related complications (TRC) is reduced with a current generation mechanical prosthesis (On-X Life Technologies/CryoLife Inc—On-X) compared with a previous generation mechanical prosthesis (St Jude Medical Inc—SJM). The study hypothesis was designed to access the null (H0) and alternative (HA) hypotheses.

## Methods

The study design of the PROSE trial was a multi-centre, randomized trial that would sequentially enrol 400 eligible patients in each group from up to 25 participating study centres (the actual enrollment centres was 28 centres) in worldwide centres incorporating Western and Developing countries. The UN Development Programme (UNDP) value for the Human Development Index (HDI) identified arbitrarily the use of 0.9 and above for Western developed countries and 0.75 and below for Developing countries. The categorization revealed essentially a 50/50 split in the total study population. A country such as China which could be considered in the transition expressed no interest in participation in the study. To ensure adequate enrolment, the number of mechanical prosthesis implants performed was the key criterion in selection of these sites. It was estimated that each centre would be able to randomize a minimum of 30–40 patients. In actual fact, the distribution of the patients per centre did not meet this anticipated distribution. The final analysis would begin approximately one year after the final patient was enrolled, resulting in study completion within five (5) years.

The patient eligibility of the trial included the inclusion and exclusion criteria. Patient eligibility was determined, and patient consent was obtained within seven (7) days before operation.

The inclusion criteria were:

1. The patient required an isolated mitral or isolated aortic prosthesis replacement. (Patients undergoing coronary artery bypass and/or concomitant repair of mitral or tricuspid valves were eligible).
2. The patient would be a candidate for receipt of a mechanical heart prosthesis.
3. The patient (or legal guardian) had signed a study-specific informed consent form agreeing to the randomization, data collection and follow-up requirements.
4. The patient could be having a re-operative procedure with the previous prosthesis explanted and the patient does not become a double prosthesis implantation patient.

The exclusion criteria were:

1. The patient was not a candidate to receive a mechanical heart prosthesis.
2. The patient already had a prosthetic valve other than the prosthesis (es) being replaced at the time of the study commencement.
3. The patient required a tricuspid replacement.
4. The patient was enrolled in another investigative study or trial.

The randomization assignment of patients in the PROSE trial eliminated potential selection biases and reduced the likelihood of disproportionate distribution of both known and unknown prognostic factors between the treatment control groups. The study personnel at each site determined the randomization assignment during surgery by opening a sequentially numbered, sealed envelope for each eligible patient. Using this envelope system, patients were randomized with equal probability with one or two treatment groups, On-X mechanical prosthesis or SJM mechanical prosthesis. The random assignment of patients would be different for both aortic and mitral position. All study personnel were blinded to the randomization schedule. A randomization log containing procedural instructions and log sheets for recording randomization information were provided to each site. Any violations of the randomization assignment were communicated to the co-ordinating centre following the discovery.

The follow-up of patients occurred at discharge, 3-months, 6-months, at 1-year and annually thereafter during the conduct of the study and the longitudinal evaluation to approximately 5-years. Data collected included information regarding adverse events as defined as the “Guidelines for reporting morbidity and mortality after cardiac valvular operations” of the Society of Thoracic Surgeons and the American Association for Thoracic Surgery (STS/AATS) [1]. The specific adverse events of thromboembolism and hemorrhage were specifically delineated. The thromboembolic events were delineated as reversible ischemic neurological deficit (RIND), major and thrombosis and were confirmed by clinical evaluation, echocardiography or computerized scans, as needed. The hemorrhagic events were all major events as defined by the guidelines inclusive of hospitalization and/or blood product transfusion as an in-patient or an outpatient. The additional follow-up included New York Heart Association (NYHA) functional classification, specific procedures and medications. The follow-up was initially conducted by telephone evaluation and contact with the attending physician as deemed necessary. If difficulty was encountered in obtaining the needed follow-up and/or complications from the information, the patient was contacted and scheduled for an office visit by the centre investigator. If this was not possible, the investigator contacted the patient’s follow-up physician to obtain the required data. The patient management was conducted by the patient’s attending physician, whether that be family physician, internist or cardiologist. The attending physician received notification that the patient was involved in the PROSE study along with recommendations with regard to target anticoagulation. The target anti-coagulation level for aortic prostheses was INR between 2.2 and 2.8 and mitral prostheses was for 2.5–3.5.

The sample size for the PROSE randomized trial was dependent upon many assumptions including a projected rate of events, the measure required to detect the difference between the treatment and control groups, the selected Type I and Type II error rates and the type of significant tests used. The sample size calculation for the PROSE study assumed a rate of 1.0% per patient for major thrombotic events with the On-X prosthesis and a rate of 2.0% per patient for the St Jude Medical prosthesis based on existing literature [2–11]. The sample size was calculated on the rate of late thromboembolic major events that was standardly reported in the literature or from regulatory trials for the prostheses.

It was assumed that the treatment group (On-X) would experience a 50% reduction in the incidence of major thromboembolic events relative to the SJM group. The 50% magnitude of major thromboembolic event reduction was considered clinically important, as well as detectable with the expected sample size. An exponential maximum likelihood test of equality of survival curves with a 0.050 one-sided significance level would have 80% power to detect the difference between a rate of 0.0100 for the On-X prosthesis and a rate of 0.0200 for the SJM prosthesis.

The data analysis will be performed with an “intent to treat” analysis, with no crossovers in the trial, actually not allowed in the trial. For the data analysis patients will be included in the treatment group in which they were assigned. By randomizing patients during surgery, deviations from the randomization assignment and the resulting of the dilution of the treatment effects would be minimized.

Linearized occurrence rates will be utilised to determine the performance of the prostheses with regard to the overall and major thromboembolic events and haemorrhage events. Kaplan–Meier analysis will also be utilised to evaluate the performance of the two prostheses with regard to freedom from thromboembolic events. A log-rank test will be utilised to validate the significance of the Kaplan–Meier analysis.

The true significance of the On-X mechanical prosthesis in reducing the incidence of thromboembolism is unknown. The current documented thromboembolic rates with the On-X prosthesis comes from the regulatory trials conducted for the Food & Drug Administration of the United States, and clinical studies [2–6]. The thromboembolic rates for the St Jude Medical prosthesis are well documented in the literature from publications over the past 20 years [7–11]. The thromboembolism rate for the On-X prosthesis was considered to be 1.0%/patient-year from the regulatory trials and that of the SJM prosthesis to be 2.0%/patient- year from the extensive publications. Final analysis of the randomized trial will be reviewed to ascertain if the observed differences are clinically important.

The Adjudication Committee of the PROSE study consisted of the Data Safety Monitoring Board (DSMB) and the co-ordinating centre for the PROSE study at the Vancouver site. The primary end-point adjudication was conducted blinded to the DSMB. This method of adjudication blinding of end-point events is the only achievable method in a heart valve prosthesis study. The PROSE study utilized Case Report Forms for collection of the data. Each PI monitored their centre for severe adverse events as defined by the STS/AATS guidelines [1]. The sponsor and each of the centres reported the serious adverse events (SAE) to the appropriate governments, as required by each country’s law for commercially distributed products.

The PROSE study was carried out according to the principals of the Helsinki Declaration. The written, informed consent for an eligible patient was required before the patient could be included in the investigational trial. The signed consent indicated that the patient agreed to accept the random assignment of the type of prosthesis, either SJM or On-X. Each of the patients indicated that he/she would adhere to the follow-up examination schedule and completing annual data collection surveys. The signed consent form also included a statement that the study data would be made available to the sponsor (On-X Life Technologies/CryoLife Inc.). The institutional IRB (University and/or hospital) representing the prospective study site reviewed and approved the investigational plan and the prospective investigator’s participation before the investigation began at the site.

The risks of valve replacement with either of these mechanical prostheses are those associated with all prosthetic replacement surgery, including thromboembolism and bleeding, which are the focus of this study. The outcome of adverse events typical of prosthesis replacement can be transient or permanent and including death. The risk of participating in the study was that patients (50% of patients) could turn out to receive a prosthesis type that was associated with more thromboembolic events (blood clots) than the other prosthesis type they could have received. The study was designed to determine which prosthesis was safer. There was no specific benefit to participating in the study. The relative safety of the two prosthesis types was unknown at this time, although both prostheses are approved for commercial use by Canadian and United States governments, and all major worldwide governments. The determination of the relative safety was the reason for the study.

The patient progress and health status were carefully monitored in patients who were involved in the study, and any complications that arose were detected and treated (if necessary) at an early stage. Knowledge gained by participation in the study could be of potential benefit to other patients. The assessment of patient information gathered in the study would provide information that would assist in identifying the optimal heart prosthesis type for a patient with varied health and heart histories. It was possible that if the clinical results for one of the heart prostheses was superior, then the patient receiving that heart prosthesis could benefit from a reduction in the potential complications of mechanical prostheses. Any information identified that would be of importance to continuing participation will be disclosed in patients in a timely fashion. The alternative to participating in this study is to have valve replacement with the prosthesis of choice selected by the patient and attending cardiologist and cardiac surgeon.

## Results

The total population for analysis in the PROSE trial was 855 patients implanted between 2003 and 2016. There were 939 patients screened for the trial. Of the trial patients—16 discontinued/withdrew and 84 were lost to follow-up. The enrollment on the PROSE trial was completed on August 31, 2016. The follow-up for the PROSE trial will complete August 2021.

The preoperative demographics and risk factors for the total population is detailed in Tables 1, 2, 3 and 4. The preoperative demographics and risk factors by aortic and mitral valve positions are detailed in the Additional file 1: Tables E1–E4.

**Table 1:** Preoperative demographics whole population

Factor	Whole study	On-X	SJM	*p* Value
Patients (N)	855	462	393	0.232*
Follow-up (ptyrs)	4078.0	2219.8	1858.3	
Age (mean—SD)	49.0–12.6	49.2–12.7	48.9–12.4	0.728
Gender (N—% male)	503–58.8	287–62.1	216–55.0	**0.036**
NYHA (N—%)				
I	69–8.1	40–8.7	29–7.4	0.091
II	298–34.8	154–33.3	144–36.6	
III	369–43.2	193–41.8	176–44.8	
IV	78–9.1	45–9.7	33–8.4	
Unknown	41–4.8	30–6.5	11–2.8	
Etiology (N—%)				
Rheumatic	344–41.6	183–41.6	161–41.6	0.861
Calcific	246–29.8	132–30.0	114–29.5	
Prosthetic Degeneration	20– 2.4	13–3.0	7–1.8	
Congenital	109–13.2	54–12.3	55–14.2	
Endocarditis	37–4.5	21–4.8	16–4.1	
Degenerative	56–6.8	30–6.8	26–6.7	
Other	15–1.8	7–1.6	8–2.1	
Primary rhythm (N—%)				
Sinus	627–75.2	338–75.1	289–75.3	0.912
Atrial fibrillation	193–23.1	105–23.3	88–22.9	
Paced	3–0.4	1–0.2	2–0.5	
Other	11–1.3	6–1.3	5–1.3	
Aortic lesion (N—%)				
Stenosis	281–56.8	141–52.6	140–61.7	**0.0499**
Regurgitation	82–16.6	42–15.7	40–17.6	
Mixed	130–26.3	84–31.3	46–20.3	
Other	2–0.4	1–0.4	1–0.4	
Mitral lesion (N—%)				
Stenosis	80–23.2	49–26.5	31–19.4	0.224
Regurgitation	68–19.7	35–18.9	33–20.6	
Mixed	195–56.5	99–53.5	96–60.0	
Other	2–0.6	2–1.1	0–0.0	
Weight in kg (mean—SD)	73.2–22.1	74.2–22.5	72.0–21.6	0.147
Height in cm (mean—SD)	166.5–11.2	167.2–11.2	165.7–11.1	0.0504
Body surface area in m^2^ (mean—SD)	1.80–0.29	1.82–0.29	1.78–0.28	**0.042**
Body mass index in kg/m^2^ (mean—SD)	26.2–7.0	26.3–7.2	26.0–6.7	0.531

*p*-value considered significant when < 0.05 and highlighted in those cases

*Test for randomization across whole trial includes an adjustment from *p* = 0.5, because the Australian cohort was randomized 2:1 not 1:1 arriving at *p* = 0.48 for the trial

**Table 2:** Preoperative and operative risk factors whole study

Factor	Whole study	On-X	SJM	*p* Value
Smoker (N—%)	303–35.4	171–37.0	132–33.6	0.301
Coronary disease in family (N—%)	145–17.0	79–17.1	66–16.8	0.907
Diabetes (N—%)	98–11.5	52–11.3	46–11.7	0.855
High cholesterol (N— %)	221–25.8	130–28.1	91–23.2	0.103
Preoperative creatinine (mean—SD)	89.8–65.1	91.7–71.6	87.6–56.8	0.360
Renal failure (N—%)	35–4.1	18–3.9	17–4.3	0.768
Hypertension (N—%)	329–38.5	176–38.1	153–38.9	0.811
History of CVA (N—%)	45–5.3	26–5.6	19–4.8	0.601
Previous endocarditis (N—%)	37–4.3	23–5.0	14–3.6	0.318
Existing COPD (N—%)	81–9.5	45–9.7	36–9.2	0.804
Immunosuppressed (N—%)	12–1.4	8–1.7	4–1.0	0.382
Peripheral vascular disease (N—%)	23–2.7	16–3.5	7–1.8	0.128
Carotid vascular disease (N—%)	45–5.3	23–5.0	22–5.6	0.696
Previous cardiac surgery (N—%)	124–14.5	66–14.3	58–14.8	0.836
Previous MI (N— %)	41–4.8	24–5.2	17–4.3	0.539
Congestive heart failure (N—%)	217–25.4	124–26.8	93–23.7	0.300
Angina (N—%)	116–13.6	60–13.0	56–14.2	0.610
Cardiogenic shock (n—%)	4–0.5	3–0.6	1–0.3	0.519
Resuscitation (N—%)	5–0.6	3–0.6	2–0.5	0.844
Ejection fraction % (mean—SD)	55.5–11.5	55.2–11.4	55.9–11.5	0.373
Preoperative status (N—%)				
Elective	657–84.0	355–84.9	302–83.0	0.757
Urgent	119–15.2	60–14.4	59–16.2	
Emergent	6–0.8	3–0.6	3–0.8	
Aortic valve percentage	502–58.7	273–59.1	229–58.2	
Concomitant procedures (N—%)	286–33.4	152–32.9	134–34.1	0.711
Intraoperative AE’s (N—%)	76–8.9	42–9.1	34–8.7	0.838

**Table 3:** Preoperative demographics for Western versus Developing worlds

Factor	Whole study	Western	Developing	*p* Value
Patients (N)	855	437	418	
Follow-up (ptyrs)	4078.0	2213.3	1864.8	
Age (mean—SD)	49.0–12.6	54.5–9.8	43.3–12.6	< 0.0001
Gender (N—% male)	503–58.8	311–71.2	192–45.9	< 0.0001
NYHA (N—%)				
I	69–8.1	64–14.6	5–1.2	< 0.0001
II	298–34.8	154–35.2	144–34.4	
III	369–43.2	152–34.8	217–51.9	
IV	78–9.1	48–11.0	30–7.2	
Unknown	41–4.8	19–4.4	22–5.3	
Etiology (N—%)				
Rheumatic	344–41.6	30–7.9	314–70.1	< 0.0001
Calcific	246–29.8	194–51.2	52–11.6	
Prosthetic degeneration	20–2.4	6–1.6	14–3.1	
Congenital	109–13.2	92–24.3	17–3.8	
Endocarditis	37–4.5	16–4.2	21–4.7	
Degenerative	56–6.8	35–9.2	21–4.7	
Other	15–1.8	6–1.6	9–2.0	
Primary rhythm (N—%)				
Sinus	627–75.2	364–87.5	263–62.9	< 0.0001
Atrial fibrillation	193–23.1	44–10.6	149–35.6	
Paced	3–0.4	1–0.2	2–4.8	
Other	11–1.3	7–1.7	4–9.6	
Aortic lesion (N—%)				
Stenosis	281–56.8	250–66.5	31–26.1	< 0.0001
Regurgitation	82–16.6	49–13.0	33–27.7	
Mixed	130–26.3	77–20.5	53–44.5	
Other	2–0.4	0–0.0	2–1.7	
Mitral lesion (N—%)				
Stenosis	80–23.2	13–26.0	67–22.7	< 0.0001
Regurgitation	68–19.7	21–42.0	47–15.9	
Mixed	195–56.5	16–32.0	179–60.7	
Other	2–0.6	0–0.0	2–0.7	
Weight in kg (mean—SD)	73.2–22.1	86.0–19.8	60.4–16.1	< 0.0001
Height in cm (mean—SD)	166.5–11.2	172.1–9.7	161.0–9.7	< 0.0001
Body surface area in m^2^ (mean—SD)	1.80–0.29	1.98–0.23	1.62–0.21	< 0.0001
Body mass index in kg/m^2^ (mean—SD)	26.2–7.0	29.1–6.6	23.3–6.1	< 0.0001

**Table 4:** Preoperative and operative risk factors for Western versus Developing worlds

Factor	Whole study	Western	Developing	*p* Value
Smoker (N—%)	303–35.4	231–52.9	72–17.2	** < 0.0001**
Coronary disease in family (N—%)	145–17.0	127–29.1	18–4.3	** < 0.0001**
Diabetes (N—%)	98–11.5	66–15.1	32–7.7	**0.0004**
High cholesterol (N—%)	221–25.8	193–44.2	28–6.7	** < 0.0001**
Preoperative creatinine (mean—SD)	89.8–65.1	98.1–91.0	83.1–28.4	**0.001**
Renal failure (N—%)	35–4.1	19–4.3	16–3.8	0.711
Hypertension (N—%)	329–38.5	243–55.6	86–20.6	** < 0.0001**
History of CVA (N—%)	45–5.3	24–5.5	21–5.0	0.743
Previous endocarditis (N—%)	37–4.3	19–4.3	18–4.3	0.989
Existing COPD (N—%)	81–9.5	61–14.0	20–4.8	** < 0.0001**
Immunosuppressed (N—%)	12–1.4	11–2.5	1–0.2	**0.003**
Peripheral vascular disease (N—%)	23–2.7	18–4.1	5–1.2	**0.009**
Carotid vascular disease (N—%)	45–5.3	26–5.9	19–4.6	0.395
Previous cardiac surgery (N—%)	124–14.5	54–12.4	71–17.0	**0.057**
Previous MI (N—%)	41–4.8	36–8.2	5–1.2	** < 0.0001**
Congestive heart failure (N—%)	217–25.4	95–21.7	122–29.3	**0.011**
Angina (N—%)	116–13.6	90–20.6	26–6.2	** < 0.0001**
Cardiogenic shock (n—%)	4–0.5	1–0.2	3–0.7	0.272
Resuscitation (N—%)	5–0.6	2–0.5	3–0.7	0.705
Ejection fraction % (mean—SD)	55.5–11.5	56.3–12.9	54.9–10.2	0.080
Preoperative Status (N—%)				
Elective	657–84.0	321–88.2	336–80.4	**0.023**
Urgent	119–15.2	40–11.0	79–18.9	
Emergent	6–0.8	3–0.8	3–0.7	
Aortic valve percentage	502–58.7	381–87.2	121–29.0	** < 0.0001**
Concomitant procedures (N—%)	286–33.4	133–30.4	153–36.7	0.051
Intraoperative AE’s (N—%)	76–8.9	56–12.8	20–4.8	** < 0.0001**

*p*-value considered significant when < 0.05 and highlighted in those cases

The total population (Table 1) of the PROSE study comprised 855 patients with On-X population 462 patients and the SJM population 393 patients. The Australian sites had 84 patients and conducted the randomization 2:1 On-X to SJM. This issue was corrected by randomization in blocked groups of 20 to force a difference of no more than 2 in the block which kept further randomization equal. The Excel random number generator was utilized to create randomization envelopes for the study. Due to the 2:1 randomization in Australia, the expected ratio for the complete population was 0.48, SJM (i.e., 410 expected of 855) to On-X (445 expected) (as noted in the footnote to Table 1). The *p* value with the Australian recruitment variance considered for the population was 0.232, indicating satisfactory randomization. Because randomization remains within statistical acceptability, this variation is not expected to affect results. This anomaly was identified after the Canadian centers commenced the study.

Table 1 identifies specific differences between the On-X and SJM prostheses populations, with a gender difference that is also reflected in the lesion distribution and body surface area but not individually in height and weight. The mean age of the total population was 49.0 years with a standard deviation of 12.6 years. The gender distribution was 58.8% male. Rheumatic valve etiology was 41.6% while calcific valvular disease was 29.8%. Sinus rhythm was present in 75.2% of patients and atrial fibrillation was present in 23.1% of patients.

The preoperative demographics for Aortic Valves (E1) and Mitral Valves (E2) revealed significant differences similar to the entire population only in the aortic position between On-X and SJM prostheses. The mean age for aortic prostheses patients was 52.3 +/− 11.4 years. Aortic patients were 13.7% rheumatic and 46.9% were calcific valvular disease. Of the aortic patients 92.4% were in sinus rhythm and only 5.6% were in atrial fibrillation. The mean age for mitral prostheses patients was 44.4 +/− 12.8 years. Mitral patients were 81.7% rheumatic and 5.0% were calcific valvular disease. Of the mitral patients 51.3% were in sinus rhythm and 47.6% were in atrial fibrillation.

The preoperative and operative risk factors for the total population are detailed in Table 2, while for aortic prostheses was detailed in Additional file 1: Table E3 and for mitral prostheses was detailed in Additional file 1: Table E4. There were no significant differences between On-X and SJM patients for all preoperative and operative risk factors.

The Western and Developing Worlds provided the most significant differences for both preoperative and operative demographics (Table 3) and for preoperative and operative risk factors (Table 4). The preoperative demographics revealed the patients in the Developing world were younger (43.3 +/− 12.6 years versus 54.5 +/− 9.8 < 0.0001), predominantly female (54.0% versus 29.0% < 0.0001), predominantly rheumatic disease (70.1% vs. 7.9% < 0.0001), and in atrial fibrillation (35.6% vs. 10.1% < 0.0001).

Aortic stenosis was more common in the Western world (66.5% vs. 26.1% < 0.0001) while aortic regurgitation more common in the Developing world (27.7% vs. 13.0% < 0.0001) (Table 3). Mixed mitral disease was more common in the Developing world (60.7% vs. 32.0% < 0.0001) while mitral regurgitation was more common in the Western world (42.0% vs. 15.9% < 0.0001) (Table 4).

The preoperative and operative risk factors for Western and Developing worlds (Table 4) revealed a complete contrast for almost all risk factors with the significant factors predominantly in the Western world. The comparative risk factors that had higher occurrence rates or measured values in the Western world population were—coronary artery disease (29.1% vs. 4.3% < 0.0001), diabetes mellitus (15.1% vs. 7.7% 0.0004), hypercholesterolemia (44.2% vs. 6.7% < 0.0001), preoperative creatinine (98.1 +/− 91.0 µmol/L vs. 82.9 +/− 28.5 µmol/L, *p* = 0.001), hypertension (55.6% vs. 20.6% < 0.0001), COPD (14.0% vs. 4.8% < 0.0001), previous myocardial infarction (8.2% vs. 1.2% < 0.0001), angina pectoris (20.6% vs. 6.2% < 0.0001). The aortic valve percentage was more common in the Western world (87.2% vs. 29.0% < 0.0001). Intraoperative adverse events were more common in the Western world (12.8% vs. 4.8% < 0.0001). Congestive heart failure, on the other hand, was more common in the Developing world (29.3% vs. 21.7% 0.011).

## Discussion

**Fig. 1:**
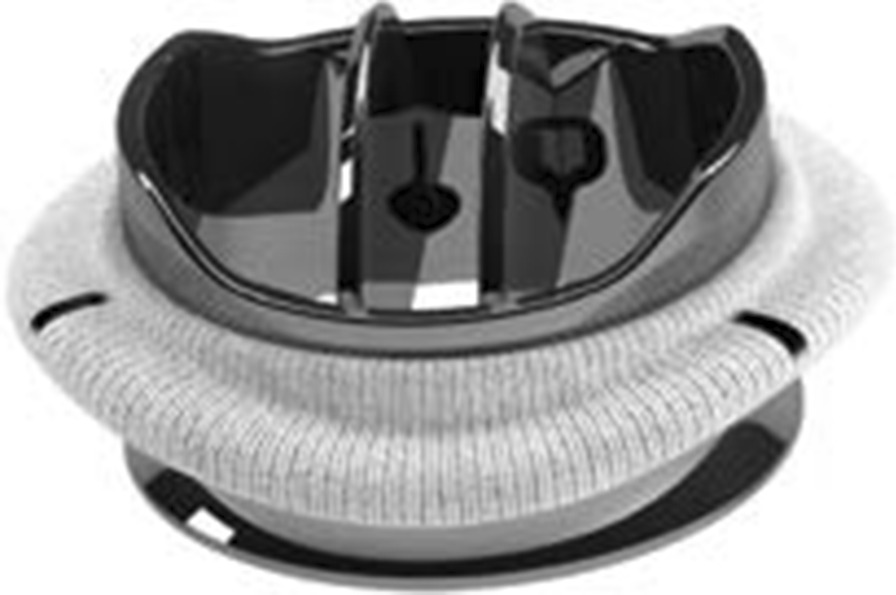
On-X aortic valve

**Fig. 2:**
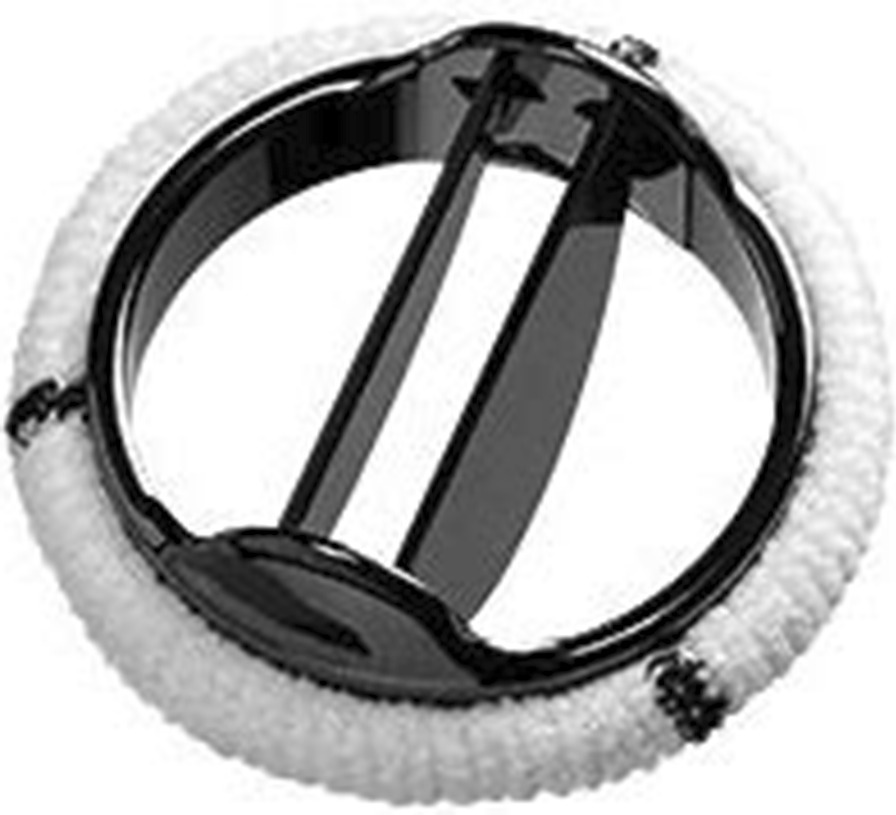
SJM aortic valve

Table 5 (Figs. 1 and 2).

**Table 5:** On-X versus SJM design comparison

Feature	On-X Valve (On-X Fig. 1)	SJM Valve (SJM Fig. 2)
Material	Pure pyrolytic carbon	Silicon-alloyed pyrolytic carbon
Sewing ring position	Supra-annular	Supra-annular
Valve position	Intra-supra-annular	Supra-annular
Pannus overgrowth protection	Yes	No
Orifice length	Longer natural length-to-diameter ratio	Shorter less than natural length-to-diameter ratio
Pivot design	Actuated by remote center of rotation	Fixed rotation point
Leakage path	Smooth through contoured pivot with set gap tolerances	Jet through angular pivot
Closing geometry	Two points at 45° from leaflet tip reducing closing velocity	Single point at tip of leaflet

### On-X specific design features (Table 5, Fig. 1)

As compared in Table 5 the On-X prosthesis is a pure pyrolytic carbon prosthesis with a supraannular sewing ring. The prosthesis design facilitates pannus protection (pannus protection was not a comparative feature of the PROSE trial). The long, flared orifice of the On-X prosthesis facilitates organized flow through the prosthesis (height-to-diameter ratio of about 0.6). The actuated pivots of the On-X prosthesis allow the leaflets to follow the blood flow through the prosthesis. The pivot purge of the On-X prosthesis facilitates the elimination of blood stasis in the prosthesis. The two-point closure of the On-X reduces the impact of leaflet closure.

### SJM specific design features (Table 5, Fig. 2)

The SJM prosthesis is made from a silicon-alloyed pyrolytic carbon that is less strong and more brittle than pure pyrolytic carbon. It also features a supra-annular sewing ring, but its orifice does not extend above and below the ring except at the pivot ears providing little barrier to pannus overgrowth. The height-to-diameter ratio of the housing is approximately 0.3. Its leaflets rotate on a fixed pivot and its closing contact points are at the tips of the leaflets resulting in a higher likelihood of cavitation.

### Limitations

The study was designed to approximate standard care outside of a trial. As such, elements were left uncontrolled and unevaluated, such as routine INR management, even though targets were provided. This will cause results analysis will be limited appropriately. Further research needs may arise from the final analyses.

## Conclusion

The completion of the long-term follow-up in eight [8] residual centres in the Developing world will provide the opportunity to evaluate the influence of prosthesis-type on major thromboembolism, thrombosis and major hemorrhage in accordance with the objectives of the PROSE trial. The influence of prosthesis-type in the Western world and the Developing world will also be evaluated for major thromboembolism, thrombosis and hemorrhage. These comparisons will be conducted by the overall population and by valve position. The PROSE study findings will afford the opportunity for comparison to the existing world literature.

## Supplementary Information

**Additional file 1.** Preoperative demographics and risk factors for aortic valve patients and mitral valve patients by valve type.

## Data Availability

The datasets used and/or analysed during the current study are available from the corresponding author on reasonable request.
