# Supplementary material for: PROSE: Prospective Randomized Trial of the On-X Mechanical Prosthesis and the St Jude Medical Mechanical Prosthesis Evaluation: Part 1(Patient Dynamics): Preoperative demographics and preoperative and operative risk factors
Source: J Cardiothorac Surg. 2021 Nov 3;16:323. doi: 10.1186/s13019-021-01632-6 (PMC8565024; doi:10.1186/s13019-021-01632-6)
Supplement: Supplementary file 1 — Additional file 1. Preoperative demographics and risk factors for aortic valve patients and mitral valve patients by valve type. [file 13019_2021_1632_MOESM1_ESM.docx]

Table E1

Preoperative Demographics for Aortic Valves

| Factor | All Aortic Patients | On-X | SJM | p-value |
| --- | --- | --- | --- | --- |
| Patients (N) | 502 | 273 | 229 |  |
| Follow-up (ptyrs) | 2519.4 | 1379.4 | 1140.0 |  |
| Age (mean - stdev) | 52.3 – 11.4 | 52.6 – 11.1 | 51.9 – 11.7 | 0.493 |
| Gender (N - % male) | 360 – 71.7 | 207 – 75.8 | 153 – 66.8 | **0.026** |
| NYHA (N - %) |  |  |  |  |
| I | 63 – 12.6 | 38 – 13.9 | 25 – 10.9 | 0.087 |
| II | 187 – 37.3 | 100 – 36.8 | 87 – 38.0 |  |
| III | 188 – 37.5 | 92 – 33.8 | 96 – 41.9 |  |
| IV | 39 – 7.8 | 24 – 8.8 | 15 – 6.6 |  |
| Unknown | 24 – 4.8 | 18 – 6.6 | 6 – 2.6 |  |
| Etiology (N - %) |  |  |  |  |
| Rheumatic | 67 – 13.7 | 35 – 13.5 | 32 – 14.0 | 0.777 |
| Calcific | 229 – 46.9 | 123 – 47.5 | 106 – 46.3 |  |
| Prosthetic Degeneration | 10 – 2.0 | 7 – 2.7 | 3 – 1.3 |  |
| Congenital | 107 – 21.9 | 53 – 20.5 | 54 – 23.6 |  |
| Endocarditis | 26 – 5.3 | 14 – 5.4 | 12 – 5.2 |  |
| Degenerative | 40 – 8.2 | 24 – 9.3 | 16 – 7.0 |  |
| Other | 9 – 1.8 | 3 – 1.2 | 6 – 2.6 |  |
| Primary Rhythm (N - %) |  |  |  |  |
| Sinus | 448– 92.4 | 242 – 92.4 | 206 – 92.4 | 0.903 |
| Atrial Fibrillation | 27 – 5.6 | 15 – 5.7 | 12 – 5.4 |  |
| Paced | 3 – 0.6 | 1 – 0.4 | 2 – 0.9 |  |
| Other | 7 – 1.4 | 4 – 1.5 | 3 – 1.3 |  |
| Aortic Lesion (N - %) |  |  |  |  |
| Stenosis | 281 – 56.8 | 141 – 52.6 | 140 – 61.7 | **0.050** |
| Regurgitation | 82 – 16.6 | 42 – 15.7 | 40 – 17.6 |  |
| Mixed | 130 – 26.3 | 84 – 31.3 | 46 – 20.3 |  |
| Other | 2 – 0.4 | 1 – 0.4 | 1 – 0.4 |  |
| Mitral Lesion (N - %) |  |  |  |  |
| Stenosis | - |  |  | NA |
| Regurgitation | - |  |  |  |
| Mixed | - |  |  |  |
| Other | - |  |  |  |
| Weight in kg (mean – stdev) | 81.3 – 21.1 | 82.6 – 20.1 | 79.7 – 22.2 | 0.483 |
| Height in cm (mean – stdev) | 170.1 – 10.2 | 171.4 – 9.8 | 168.6 – 10.6 | 0.148 |
| Body Surface Area in m^2^ (mean – stdev) | 1.91 – 0.27 | 1.94 – 0.25 | 1.89 – 0.27 | **0.032** |
| Body Mass Index in kg/m^2^ (mean – stdev) | 28.0 – 6.9 | 28.0 – 6.5 | 28.0 – 7.0 | 1.000 |

Table E2

Preoperative Demographics for Mitral Valves

| Factor | All Mitral Patients | On-X | SJM | p-value |
| --- | --- | --- | --- | --- |
| Patients (N) | 353 | 189 | 164 |  |
| Follow-up (ptyrs) | 1166.6 | 625.9 | 540.7 |  |
| Age (mean - stdev) | 44.4 – 12.8 | 44.1 – 13.3 | 44.8 – 12.2 | 0.609 |
| Gender (N - % male) | 143 – 40.5 | 80 – 42.3 | 63 – 38.4 | 0.457 |
| NYHA (N - %) |  |  |  |  |
| I | 6 – 1.7 | 2 – 1.1 | 4 – 2.4 | 0.470 |
| II | 111 – 31.4 | 54 – 28.6 | 57 – 34.8 |  |
| III | 181 – 51.3 | 101 – 53.4 | 80 – 48.8 |  |
| IV | 39 – 11.1 | 21 – 11.1 | 18 – 11.0 |  |
| Unknown | 15 – 4.3 | 10 – 5.3 | 5 – 3.0 |  |
| Etiology (N - %) |  |  |  |  |
| Rheumatic | 277 – 81.7 | 148 – 81.8 | 129 – 81.6 | 0.811 |
| Calcific | 17 – 5.0 | 9 – 5.0 | 8 – 5.1 |  |
| Prosthetic Degeneration | 10 – 3.0 | 6 – 3.3 | 4 – 2.5 |  |
| Congenital | 2 – 0.6 | 1 – 0.6 | 1 – 0.6 |  |
| Endocarditis | 11 – 3.2 | 7 – 3.9 | 4 – 2.5 |  |
| Degenerative | 16 – 4.7 | 6 – 3.3 | 10 – 6.3 |  |
| Other | 6 – 1.8 | 4 – 2.2 | 2 – 1.3 |  |
| Primary Rhythm (N - %) |  |  |  |  |
| Sinus | 179 – 51.3 | 96 – 51.1 | 83 – 50.6 | 0.982 |
| Atrial Fibrillation | 166 – 47.6 | 90 – 47.9 | 76 – 46.3 |  |
| Paced | 0 – 0.0 | 0 – 0.0 | 0 – 0.0 |  |
| Other | 4 – 1.1 | 2 – 1.1 | 2 – 1.3 |  |
| Aortic Lesion (N - %) |  |  |  |  |
| Stenosis |  |  |  |  |
| Regurgitation |  |  |  |  |
| Mixed |  |  |  |  |
| Other |  |  |  |  |
| Mitral Lesion (N - %) |  |  |  |  |
| Stenosis | 80 – 23.2 | 49 – 26.5 | 31 – 19.4 | 0.224 |
| Regurgitation | 68 – 19.7 | 35 – 18.9 | 33 – 20.6 |  |
| Mixed | 195 – 56.5 | 99 – 53.5 | 96 – 60.0 |  |
| Other | 2 – 0.6 | 2 – 1.1 | 0 – 0.0 |  |
| Weight in kg (mean – stdev) | 62.1 – 18.2 | 62.5 – 20.4 | 61.6 – 15.5 | 0.645 |
| Height in cm (mean – stdev) | 161.7 – 10.5 | 161.5 – 10.5 | 161.8 – 10.6 | 0.790 |
| Body Surface Area in m^2^ (mean – stdev) | 1.64 – 0.24 | 1.65 – 0.26 | 1.64 – 0.22 | 0.699 |
| Body Mass Index in kg/m^2^ (mean – stdev) | 23.7 – 6.5 | 23.9 – 7.4 | 23.4 – 5.2 | 0.470 |

Table E3

Preoperative and Operative Risk Factors for Aortic Valves

| Factor | Aortic Valves | On-X | SJM | p-value |
| --- | --- | --- | --- | --- |
| Smoker (N - %) | 233 – 46.4 | 132 – 48.4 | 101 – 44.1 | 0.336 |
| Coronary Disease in Family (N - %) | 113 -22.5 | 59 – 21.6 | 54 – 23.6 | 0.593 |
| Diabetes (N - %) | 74 – 14.7 | 38 – 13.9 | 36 – 15.7 | 0.571 |
| High Cholesterol (N - %) | 198 – 39.4 | 116 – 42.5 | 82 – 35.8 | 0.126 |
| Preoperative Creatinine (mean – SD) | 89.0 – 75.1 | 99.0 – 93.8 | 86.2 – 44.4 | 0.059 |
| Renal Failure (N -%) | 24 – 4.8 | 14 – 5.1 | 10 – 4.4 | 0.714 |
| Hypertension (N - %) | 259 – 51.6 | 144 – 52.7 | 115 – 50.2 | 0.577 |
| History of CVA (N - %) | 27 – 5.4 | 19 – 7.0 | 8 – 3.5 | 0.084 |
| Previous Endocarditis (N - %) | 26 – 5.2 | 16 – 5.9 | 10 – 4.4 | 0.452 |
| Existing COPD (N - %) | 57 – 11.4 | 30 – 11.0 | 27 – 11.8 | 0.779 |
| Immunosuppressed (N - %) | 11 – 2.2 | 7 – 2.6 | 4 – 1.7 | 0.493 |
| Peripheral Vascular Disease (N - %) | 18 – 3.6 | 13 – 4.8 | 5 – 2.2 | 0.120 |
| Carotid Vascular Disease (N - %) | 27 – 5.4 | 17 – 6.2 | 10 – 4.4 | 0.374 |
| Previous Cardiac Surgery (N - %) | 43 – 8.6 | 23 – 8.4 | 20 – 8.7 | 0.905 |
| Previous MI (N - %) | 36 – 7.2 | 20 – 7.3 | 16 – 7.0 | 0.897 |
| Congestive Heart Failure (N - %) | 108 – 21.5 | 61 – 22.3 | 47 – 20.5 | 0.625 |
| Angina (N - %) | 107 – 21.3 | 53 – 19.4 | 54 – 23.6 | 0.253 |
| Cardiogenic Shock (n - %) | 2 – 0.4 | 2 – 0.7 | 0 – 0.0 | 0.205 |
| Resuscitation (N - %) | 1 – 0.2 | 1 – 0.4 | 0 – 0.0 | 0.338 |
| Ejection Fraction % (mean – SD) | 56.0 – 12.4 | 55.6 – 12.3 | 56.4 – 12.5 | 0.472 |
| Preoperative Status (N - %) |  |  |  |  |
| Elective | 369 – 85.8 | 203 – 88.3 | 166 – 83.0 | 0.275 |
| Urgent | 58 – 13.5 | 26 – 11.3 | 32 – 16.0 |  |
| Emergent | 3 – 0.7 | 1 – 0.4 | 2 – 1.0 |  |
| Concomitant Procedures (N - %) | 144 – 28.7 | 72 – 26.4 | 72 – 31.4 | 0.218 |
| Intraoperative AE’s (N - %) | 56 – 11.1 | 32 – 11.7 | 24 – 10.5 | 0.671 |

Table E4

Preoperative and Operative Risk Factors for Mitral Valves

| Factor | Mitral Valves | On-X | SJM | p-value |
| --- | --- | --- | --- | --- |
| Smoker (N - %) | 70 – 19.8 | 39 – 20.6 | 31 – 18.9 | 0.690 |
| Coronary Disease in Family (N - %) | 32 – 9.1 | 20 – 10.6 | 12 – 7.3 | 0.282 |
| Diabetes (N - %) | 24 – 6.8 | 14 – 7.4 | 10 – 6.1 | 0.629 |
| High Cholesterol (N - %) | 23 – 6.5 | 14 – 7.4 | 9 – 5.5 | 0.471 |
| Preoperative Creatinine (mean – SD) | 86.0 – 50.7 | 83.1 – 25.8 | 89.2 – 68.9 | 0.260 |
| Renal Failure (N -%) | 11 – 3.1 | 4 – 2.1 | 7 – 4.3 | 0.236 |
| Hypertension (N - %) | 70 – 19.8 | 32 – 16.9 | 38 – 23.2 | 0.139 |
| History of CVA (N - %) | 18 – 5.1 | 7 – 3.7 | 11 – 6.7 | 0.202 |
| Previous Endocarditis (N - %) | 11 – 3.1 | 7 – 3.7 | 4 – 2.4 | 0.482 |
| Existing COPD (N - %) | 24 – 6.8 | 15 – 7.9 | 9 – 5.5 | 0.372 |
| Immunosuppressed (N - %) | 1 – 0.3 | 1 – 0.5 | 0 – 0.0 | 0.365 |
| Peripheral Vascular Disease (N - %) | 5 – 1.4 | 3 – 1.6 | 2 – 1.2 | 0.751 |
| Carotid Vascular Disease (N - %) | 18 – 5.1 | 6 – 3.2 | 12 – 7.3 | 0.081 |
| Previous Cardiac Surgery (N - %) | 82 – 23.2 | 43 – 22.8 | 39 – 23.8 | 0.825 |
| Previous MI (N - %) | 5 – 1.4 | 4 – 2.1 | 1 – 0.6 | 0.233 |
| Congestive Heart Failure (N - %) | 109 – 30.9 | 63 – 33.3 | 46 – 28.0 | 0.283 |
| Angina (N - %) | 9 – 2.5 | 7 – 3.7 | 2 – 1.2 | 0.137 |
| Cardiogenic Shock (n - %) | 2 – 0.6 | 1 – 0.5 | 1 – 0.6 | 0.899 |
| Resuscitation (N - %) | 4 – 1.1 | 2 – 1.1 | 2 – 1.2 | 0.930 |
| Ejection Fraction % (mean – SD) | 55.0 – 10.3 | 54.7 – 10.5 | 55.4 – 10.1 | 0.525 |
| Preoperative Status (N - %) |  |  |  |  |
| Elective | 288 – 81.8 | 152 – 80.9 | 136 – 82.9 | 0.822 |
| Urgent | 61 – 17.3 | 34 – 18.1 | 27 – 16.5 |  |
| Emergent | 3 – 0.8 | 2 – 1.1 | 1 – 0.6 |  |
| Concomitant Procedures (N - %) | 130 – 36.8 | 68 – 36.0 | 62 – 37.8 | 0.727 |
| Intraoperative AE’s (N - %) | 19 – 5.4 | 10 – 5.3 | 9 – 5.5 | 0.934 |
